# The Summer of Seventy-Six—*Legionella pneumophila* Monologue

**DOI:** 10.3201/eid2307.160546

**Published:** 2017-07

**Authors:** Arvind Valavane, Rama Chaudhry

**Affiliations:** All India Institute of Medical Sciences, New Delhi, India

**Keywords:** Legionella pneumophila, bacteria, Legionnaires’ disease, learning activity, awareness, prevention, developing countries, monologue

The scientific community has understood that the microbes around and within us are the driving force of our life (*1*). However, the next generation is being brought up with a fear of encountering microorganisms. This generation has developed a deeply rooted view that all microorganisms are dangerous. Moreover, anthropocentric investigations have created an unrealistic view of the microorganisms in nature (*2*). Only a negligible percentage of the known microorganisms cause disease in humans, and we have created a mess in handling them.

We believe that the perspective of the next generation toward microorganisms should be changed. Activities involving creation of bacterial monologues (to think from microbes’ perspective) have been shown to enhance the conceptual understanding of key topics in microbiology and improvement in the attitude of students toward science (*3*). Use of such methods would inculcate the right interest among the next generation and teach them to respect nature.

We present a poem called *The Summer of Seventy-Six*, which deliberates about the pathogen turned environmental bacterium *Legionella pneumophila,* by walking in its shoes.

**L**earning my native dance, I
**E**njoyed the filmy cover, until you
**G**ave me a chance to
**I**nvade an unclean tower.
**O**n the summer of seventy-six, I got a
**N**ame, only to sing some veterans’ fame,
**E**very corner I took the blame,
**L**ike you never played the vexing game.
**L**ittle aerosols made the incident and your
**A**iling was a mere accident.

**P**rotozoans are my natural host,
***N****aegleria* makes a happy toast.
**E**ven in hot springs I can thrive,
**U**nder the soil I can jive.
**M**acrophages are my motel room,
**O**n my way I make them bloom,
**P**revention stops the moody gloom,
**H**auling me before the boom.
**I**n a way I am a pathogen, popping your
**L**ungs’ oxygen, but you clearly know, I am
**A**ffected by heat, copper, and a halogen.

Human-made modifications of nature are the cause for troubles of humanity, and if we embark on disturbing nature we should be ready to pay for it. One classical example of our carelessness was reflected at the American Legion Convention held in 1976 in Philadelphia, Pennsylvania, USA, which led to the discovery of genus *Legionella*. It was a team of scientists, led by Dr. Joseph McDade from the Centers for Disease Control (Atlanta, GA, USA), that isolated the bacterium from infected lung tissues in the wake of the initial outbreak, which later led to determination of the source (*4*). This team even provided answers for some retrospective unsolved respiratory illness.

Artificial water distribution systems are the main reservoirs of *Legionella* species, apart from natural freshwater sources and soil (*5*). The list of species identified in the genus *Legionella* keeps increasing day by day, and more than half of them have been implicated in human illness (*6*). The infection is considered preventable because person-to-person transmission of *Legionella* spp. has never been reported, except for 1 recent probable case (*7*).

In spite of advances in communication and technology, there is a vast gap among the industrialized and developing nations in terms of public health safety regarding this bacterium. When industrialized nations are considerably monitoring *Legionella* spp., developing nations are completely out of the picture (*8**,**9*). Moreover, considering the increasing tourism industry among developing nations, we should keep in mind that travel-associated Legionnaires’ disease is not uncommon (*10*).

Thus, creating awareness among developing nations, collaboration on research projects for evidence-based learning, exchange of resources, and sharing of knowledge should happen among us to coexist with this bacterium. After all, we are breathing in its world and not the other way around!
